# Anti-ADAMTS13 Autoantibodies: From Pathophysiology to Prognostic Impact—A Review for Clinicians

**DOI:** 10.3390/jcm12175630

**Published:** 2023-08-29

**Authors:** Cristina Dainese, Federica Valeri, Benedetto Bruno, Alessandra Borchiellini

**Affiliations:** 1 Regional Centre for Hemorrhagic and Thrombotic Diseases, AOU Città Della Salute e Della Scienza, 10126 Turin, Italy; fvaleri@cittadellasalute.to.it (F.V.); aborchiellini@cittadellasalute.to.it (A.B.); 2 Division of Hematology, AOU Città Della Salute e Della Scienza and University of Turin, 10124 Turin, Italy; benedetto.bruno@unito.it; 3 Department of Molecular Biotechnology and Health Sciences, University of Turin, 10124 Turin, Italy

**Keywords:** thrombotic thrombocytopenic purpura, Moskowitz syndrome, ADAMTS13 autoantibodies, ADAMTS13 inhibitors

## Abstract

Thrombotic thrombocytopenic purpura (TTP) is a fatal disease in which platelet-rich microthrombi cause end-organ ischemia and damage. TTP is caused by markedly reduced ADAMTS13 (a disintegrin and metalloproteinase with a thrombospondin type 1 motif, member 13) activity. ADAMTS13 autoantibodies (autoAbs) are the major cause of immune TTP (iTTP), determining ADAMTS13 deficiency. The pathophysiology of such autoAbs as well as their prognostic role are continuous objects of scientific studies in iTTP fields. This review aims to provide clinicians with the basic information and updates on autoAbs’ structure and function, how they are typically detected in the laboratory and their prognostic implications. This information could be useful in clinical practice and contribute to future research implementations on this specific topic.

## 1. Introduction

Thrombotic thrombocytopenic purpura (TTP) is a rare hematological disorder caused by a deficiency in the enzymatic function of a member of the disintegrin and metalloprotease with thrombospondin-type motifs family, an enzyme called ADAMTS13, which is synthesized primarily in the liver and, in limited quantities, by vascular endothelial cells, megakaryocytes, platelets, glomerular podocytes and glial cells. ADAMTS13 binds soluble von Willebrand Factor (VWF) and interacts with endothelium-anchored Ultra-Large VWF Multimers (ULVWFMs), resulting in the cleavage of ULVWFM strings or bundles to regulate their interaction with platelets, thus preventing the formation of blood clots in normal circulation. In patients with immune TTP (iTTP), ADAMTS13 activity is significantly reduced due to the binding of anti-ADAMTS13 autoantibodies (autoAbs) to the metalloprotease. Consequently, ULVWFMs remain uncleaved in circulation, forming platelet-rich thrombi in the microvessels under conditions of high shear stress [1]. The mechanisms by which these autoAbs inhibit ADAMTS13 enzymatic function are not fully understood, and in recent years many scientific efforts have been made to improve our knowledge on this specific topic.

## 2. Anti-ADAMTS13 Autoantibodies Pathophysiology: Production, Structure and Function

As in other autoimmune disorders, iTTP is characterized by a loss of tolerance resulting in a shift to autoimmunity [2]. Antigens derived from ADAMTS13 molecules, processed by dendritic cells, activate cross-reactive naïve CD41 T cells, which, in turn, differentiate into autoreactive effector CD41 T cells [3,4]. Autoreactive B cells recirculate into the germinal center (GC) of secondary lymph nodes, stimulated by antigens and auto reactive T helper cells and differentiate into autoAb-producing plasma cells or long-lived memory B cells [5]. Shin and colleagues performed an analysis of B cell subsets and circulating follicular T helper (cfTh) cell changes in iTTP [2]. A decreased number of post-GC memory B cells, an increased number of plasma blasts and a reduction of cfTh compared to healthy controls were found in the acute phase of iTTP. Furthermore, the authors of that study described an association between higher plasma blasts and higher ADAMTS13 autoAbs levels, with a trend toward reduced ADAMTS13 antigen levels. The same group also demonstrated that, in asymptomatic patients that underwent an ADAMTS13 relapse prior to preemptive therapy with rituximab (RTX), a significantly increased naïve B cell population, a global decrease in all memory subsets and a trend toward increased plasma blasts were present.

The autoimmune response against ADAMTS13 is polyclonal and heterogeneous [6,7]. A study demonstrated that ADAMTS13 autoAbs are primarily composed of immunoglobulin G (IgG), approximately 90% of which are of the IgG4 subtype [8]. In the cases presenting with detectable IgG4 autoAbs, IgG4 were found alone in 33% of the cases and with other IgG subtypes in 67%. The second most frequent subtype detected was IgG1 (52%), followed by IgG2 (50%) and IgG3 (33%). None of these subtypes were detected alone. Only 10–20% of the patients presented with autoAbs of IgA and IgM classes [8].

**Figure 1:**
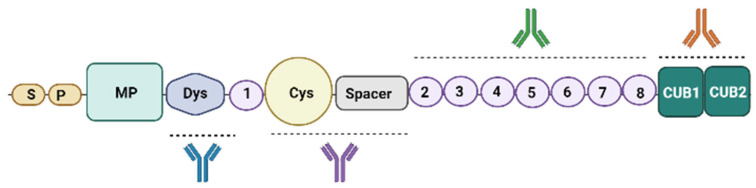
ADAMTS13 enzyme structure and major autoAbs binding sites. ADAMTS13 is a multidomain metalloprotease consisting of a signal peptide domain (S), a short propeptide domain (P), a metalloprotease (M) domain, a disintegrin-like (Dis) domain, a first thrombospondin type 1 (TSP1) repeat, a cysteine-rich (Cys) domain and a Spacer domain. It has seven additional thrombospondin type 1 repeats (TSP2-8) and two CUB domains (CUB1-2) [13]. Major epitope binding sites are the Cys-Spacer domain, the CUBs domain (see main text), the Dys domain and the TSP fragments and domains.

Several scientific groups are working to better understand which epitope/epitopes of ADAMTS13 these autoAbs recognize and bind to. Figure 1 proposes a simplified version of the ADAMTS13 structure. The physiologic functions of most of the domains are unknown. The inhibition or depletion of ADAMTS13 activity may be attributable to various mechanisms, depending on the epitope bound by the autoAbs [9]. ADAMTS13 circulates in a folded conformation through an S-CUB interaction, which is disrupted upon binding to its substrate, VWF or to opening antibodies, which allosterically activate ADAMTS13. Thus, the open conformation of ADAMTS13 induced by autoAbs is considered a hallmark of iTTP [10,11,12].

Several authors have demonstrated that a major binding site for autoAbs in iTTP is the cysteine-rich/Spacer (CS) domain [14,15,16,17]. Klaus and colleagues, by inducing the expression of a series of ADAMTS13 domains in E. coli, evaluated the reactivity of purified recombinant fragments with ADAMTS13 autoAbs from 25 patients with iTTP in vitro [18]. All the plasma samples contained autoAbs directed against the CS domain. AutoAbs reacting exclusively with the CS domain were found in 12% of plasmas, underscoring the importance of this region for the functional activity of ADAMTS13. In 64% of the plasma samples, autoAbs reacted with the two CUB domains, with the TSP-1 repeat compound fragment and the TSP-1 domain in 56%. Less frequently, autoAbs reacted with TSP1 repeats 2 to 8 (28%). Unexpectedly, autoAbs reacting with the propeptide region were found in 20% of the plasmas samples. These results indicate that, even though ADAMTS13 autoAbs react with multiple protease domains, the CS domain is consistently involved in antibody reactivity. Also, Osertag and colleagues cloned ADAMTS13 autoAbs using phage display and characterized them with respect to genetic origin, inhibition of ADAMTS13 activity and epitope specificity. It was noted that both autoAbs directed against the amino-terminal domains and those requiring the ADAMTS13 CS domains for binding inhibited proteolytic activity, while those solely targeting carboxy-terminal domains were non-inhibitory [19]. A different group isolated ADAMTS13 autoAbs sequences from the peripheral blood mononuclear cells of iTTP patients. Three IgG ADAMTS13 autoAbs were cloned (TTP73–1 and TR8C11 binding to the CS domains, ELH2–1 recognizing the T2–T3 domains). Interestingly, none of the autoAbs had inhibitory activity and all three antibodies recognized cryptic epitopes, in accordance with the hypothesis that the conformation of ADAMTS13 is open during acute iTTP [12]. Thomas and colleagues also found that almost 97% of iTTP episodes had IgG recognizing ADAMTS13 N-terminal domains and S domain. In the same study, functional analyses were performed on IgG from 43 patients and revealed that inhibitory IgG was limited to anti-S domain autoAbs [20]. AutoAbs with no detectable inhibitory action were found in 35% of patients, while 74% of patients had autoAbs with inhibitory function that was insufficient to account for the severe deficiency state, suggesting a possible alternative pathogenic mechanism. A multicenter European study determined anti-ADAMTS13 immunoprofiles based on the presence or absence of anti-M, anti-Dys, anti-CS, anti-T2-T5, anti-T6-T8 and anti-CUB1-2 autoAbs in a large cohort of both acute and remission iTTP plasma and serum samples (365 samples from 213 iTTP patients) [21]. Three main profiles were identified: only anti-CS autoAbs (profile 1); anti-CS and anti-CUB1-2 AutoAbs (profile 2); and anti-Dys, anti-CS, anti-T2-T5, anti-T6-T8 and anti-CUB1-2 autoAbs (profile 3). In both acute and remission phases, profile 1 was the dominant immunoprofile, suggesting that anti-CS autoAbs are the first to reappear or are the ones that persist during remission, while the other domain-specific autoAbs mainly appear in the acute phase. A similar analysis was performed on a Japanese iTTP cohort: more than 70% of patients had anti-CS autoAbs, in agreement with the Caucasian cohorts, but the Japanese cohort only showed one dominant immunoprofile, profile 1, with only autoAbs against the CS domain [22]. 

Increasing knowledge on autoAb immunoprofiles might support the improvement of targeted therapies for better iTTP patient management. For example, if a patient has only anti-S autoantibodies, an rADAMTS13 variant, mutated in the S domain, could be used to escape the binding of anti-S autoAbs [23,24]. 

How do ADAMST13 autoAbs work? ADAMTS13 deficiency may manifest with reduced activity and/or a reduction in circulating antigen depending on the autoAbs mechanism. Thus, the autoAbs response to ADAMTS13 includes neutralizing and/or non-neutralizing antibodies. Neutralizing autoAbs block the proteolytic activity of ADAMTS13 towards VWF, primarily reducing the enzymatic function of the molecule, while non-neutralizing autoAbs may contribute to increasing ADAMTS13 clearance or interfere with ADAMTS13 interaction with cells or other plasma proteins. As previously reported, anti-CS domain autoAbs mainly inhibit the function of ADAMTS13 by targeting the S domain, which plays a key role in VWF binding [25]. Thus, the inhibition of ADAMTS13’s function has long been thought to be the major cause of ADAMTS13 deficiency in iTTP. However, many iTTP plasma samples do not contain inhibitory ADAMTS13 autoAbs and, on the other hand, ADAMTS13 antigen levels can be severely decreased even in the presence of inhibitory ADAMTS13 autoAbs. Hence, some authors have suggested that ADAMTS13 clearance rather than ADAMTS13 inhibition could be the major pathogenic cause of ADAMTS13 deficiency in iTTP [20]. A possible enhanced clearance pathway could be the formation of ADAMTS13 antigen–antibody immune complexes (ICs), described in acute iTTP and during remission [26,27,28]. The clearance of IgG containing ICs occurs primarily in the liver, through both Fc receptor-dependent and receptor-independent mechanisms [29,30,31,32,33]. Complement also plays an important role in the elimination of IC, with C3b binding keeping ICs soluble [30]. Erythrocytes bind these opsonized ICs in the circulation via C3b receptors and expose them to tissue macrophages for elimination [31].

Recently, Underwood and colleagues demonstrated the enhanced rate of ADAMTS13 clearance and how this appears to be the major mechanism of reduced ADAMTS13 activity during plasma exchange (PEX) [34]. The authors observed that, at presentation, the vast majority of patients included in the study had ADAMTS13 antigen levels of <10%, suggesting a major contribution of ADAMTS13 clearance to the deficiency state. After the first PEX, both ADAMTS13 antigen and activity levels increased similarly, while the ADAMTS13 autoAb titer decreased in all patients, revealing ADAMTS13 inhibition to be a modest modifier of ADAMTS13 function. Analysis of ADAMTS13 antigen levels between consecutive PEX treatments revealed that the rate of ADAMTS13 clearance in more than half of patients analyzed was 4- to 10-fold faster than the estimated normal rate of clearance, again supporting the hypothesis that ADAMTS13 clearance mediated by autoAbs plays a major role in iTTP. The real picture is probably much more complicated, and it is possible that more mechanisms could act simultaneously in inducing ADAMTS13 deficiency, just as it is possible that different mechanisms could be activated in the acute phase and in case of recurrence.

In summary, the development of ADAMTS13 autoAbs is the result of an immune imbalance involving B cells, cTfh cells and plasmablasts, leading to a polyclonal autoimmune response. Most ADAMTS13 autoAbs belong to the IgG family and recognize the CS domain of the ADAMTS13 molecule. One matter of debate is the main function of autoAbs, neutralizing or non-neutralizing, in the enhancement of ADAMTS13 clearance. Increasing knowledge about the structure, function and specific epitope recognition of ADAMTS13 autoAbs not only helps to better understand iTTP pathophysiology but, as seen here, could have important clinical-therapeutic implications in the future, especially with the advent of new molecules.

## 3. Anti-ADAMTS13 Autoantibodies Detection

A test of ADAMTS13 activity is required to confirm TTP diagnosis. Then, in order to differentiate iTTP from congenital TTP (cTTP), the identification of ADAMTS13 autoAbs is mandatory. Diagnostic samples for ADAMTS13 activity and autoAbs testing should be collected prior to treatment [35]. Severe deficiency is defined as ADAMTS13 activity < 10 IU/dL (or <10% of normal values) [36,37]. Rare cases of iTTP with normal ADAMTS13 activity have been reported. This is attributed to the disassociation of neutralizing autoAbs from ADAMTS13 in vitro, allowing the recovery of activity in vitro [38]. False-negative autoAbs testing can occur with low-antibody titers or if autoAbs are highly bound in antigen–antibody complexes. This may be diagnostically misleading, masking the underlying immune mechanism of the disorder; it is thus crucial to differentiate iTTP from cTTP [39].

Bethesda assays are used to detect and titer neutralizing autoAbs. Test plasma is heat-treated to inactivate any ADAMTS13 still present, leaving autoAbs in the plasma intact. One volume of heat-treated plasma is added to one volume of normal pooled plasma (NPP), the source of ADAMTS13 in the assay. A separate control mixture is prepared, comprising equal volumes of NPP and buffer; in this way, both tubes begin the procedure with identical levels of ADAMTS13. Then, both tubes are incubated for 30 to 120 min, depending on the protocol, to permit the formation of antigen–antibody complexes. The ADAMTS13 activity of the test and control mixtures is then measured in a functional assay and the residual ADAMTS13 activity of the test sample is calculated as a percentage of that of the control sample subjected to identical incubation conditions. One Bethesda unit (BU) is an inhibitor titer that decreases the residual activity to 50% of the expected value. Performing a dilution series allows for titer determination. Rather than wait for the result on just the 1 + 1 dilution, it is common to perform the assay on a range of dilutions in the first instance, then correcting for the dilution factor [40]. Some authors suggest that an incubation period of at least 2 h and not immediate incubation is the required time for detecting inhibitory anti-ADAMTS13 antibodies [41]. It is important to note that the Bethesda-like detection of ADAMTS13 inhibitors also shows variability, dependent on the analytical technique.

The tests commonly referred to as ADAMTS13 autoAbs tests utilize enzyme-linked immunosorbent assay (ELISA) [35]. ELISA identifies all autoAbs, regardless of neutralizing or non-neutralizing activities. For this reason, IgG ELISA is more sensitive for iTTP, but less specific, since non-neutralizing autoAbs have been reported in a small percentage of healthy individuals and patients with other autoimmune disorders (e.g., systemic lupus erythematosus (SLE) or antiphospholipid antibody syndrome (APS)) [42,43,44]. For this assay, the microplate walls are supplied coated with rADAMTS13 to capture autoAbs. The first step of the assay is the addition of calibration and control plasmas containing ADAMTS13 autoAbs, as well as diluted test plasmas potentially containing ADAMTS13 autoAbs. After incubation, residual plasma is washed off, and an anti-human IgG antibody conjugated to the enzyme horseradish peroxidase (HRP) is added. The amount that binds is proportional to the amount of anti-ADAMTS13 antibody captured by the rADAMTS13. After incubation, excess conjugate is washed off, and a colorless HRP substrate, 3,3′,5,5′-tetramethylbenzidine (e.g., TMB or OPD), is added that reacts with HRP to generate a blue-colored product. After incubation, the reaction is stopped with sulfuric acid to stabilize color development, generating a clear yellow color due to TMB oxidation. Color intensity is proportional to bound conjugate and, hence, to the ADAMTS13 autoAbs level. Different ELISA set-ups are used in clinical and research laboratories, which vary in the presentation of rADAMTS13 and the type of detection autoAbs [40]. One commonly used ELISA assay is available from Technoclone, and detects human immunoglobulin (Ig) G against ADAMTS13. One significant limitation of this assay, however, is the potential to detect non-ADAMTS13 antibodies that may be present in patients with general auto-immune conditions, particularly if high levels of such antibodies are present. Dekimpe and colleagues assessed the influence of different rADAMTS13 presentation and autoAbs detection approaches in ELISA [45]. The authors concluded that although different methods of rADAMTS13 presentation for ADAMTS13 autoAb level determination correlate strongly, the detection of low ADAMTS13 autoAb levels can depend on the method of rADAMTS13 presentation.

ELISA-positive/Bethesda-negative results have been described in recovered iTTP patients who showed both ELISA- and Bethesda-positive autoAbs results at the time of acute iTTP diagnosis, confirming that inhibition is not necessarily the primary effect of some ADAMTS13 autoAbs. Thus, using only the Bethesda assay will lead to the under-detection of ADAMTS13 autoAbs [46] and generally cannot detect antibody titers below 0.5 BU. Bethesda and ELISA assays do not allow discrimination between free antibody and antibody bound to ADAMTS13 in circulating immune complexes in vivo. In conclusion, the literature data suggest that ELISA should be the preferred antibody assay at iTTP presentation, but that it requires supplementation with Bethesda assay to demonstrate the inhibitory function of the autoantibodies [46].

## 4. Anti-ADAMTS13 Autoantibodies and Prognostic Role

Together with ADAMTS13 activity and other potential prognostic markers, several authors have evaluated whether and what role ADAMTS13 autoAbs could have in a prognostic sense, considering both the impact on severity and mortality, and on the recurrence risk. Ferrari et al. observed that patients included in their study were less likely to survive their first iTTP event if they had IgG1 and very low or undetectable IgG4 levels plus higher titers of other classes of ADAMTS13 autoAbs (particularly IgA), suggesting that an immune response characterized by high levels of IgG4 could, at least partially, predict a more treatable form of iTTP [8]. The authors also investigated the possibility of an association between IgG subclasses and relapse and found that high levels of IgG4 with undetectable IgG1 was significantly associated with a trend towards iTTP recurrence. A different Italian group found that during acute-phase iTTP IgA represented the Ig class which most strongly associated with clinical severity (estimated in this study by the number of platelets at presentation) [47]. The authors suggested that IgA could contribute to the severity of the clinical manifestations by activating complement system through the mannose-binding lectin pathway, thus increasing complement-mediated inflammation [48]. In the same study, the IgG class and IgG subclass were also found to be predictive of the severity of the acute iTTP episode, as high IgG titers were associated with a higher number of PEXs and IgG1 and IgG3 were the classes most strongly associated with the clinical severity of acute-phase disease. Different authors have also suggested that high-titer inhibitors are associated with delayed response to PEX and refractory disease [49,50,51]. Alwan and colleagues demonstrated in a registry-based retrospective study that patients with ADAMTS13 autoAbs levels in the upper quartile had a mortality rate more than three times higher than that of patients with ADAMTS13 autoAbs in the lowest quartile. When comparing the upper and lower quartiles, those in the upper quartile were also more likely to have a raised troponin and a reduced Glasgow Coma Scale (GCS), and required a longer period of PEX to achieve platelet count normalization [52]. Another prospective multicenter study was conducted to assess the prognostic value of inhibitory ADAMTS13 autoAbs. It was found that patients with no detectable inhibitors usually displayed a more rapid and durable response to treatment, whereas patients with detectable inhibitors had a delayed improvement in ADAMTS13 activity and platelet count recovery, hence requiring significantly higher volumes of plasma to achieve durable complete remission [53]. In the same study, death was only observed in patients with an intermediate or high ADAMTS13 inhibitor titer at diagnosis, while all patients with a low inhibitor titer evolved favorably. This suggests that the strength of ADAMTS13 autoAbs may be associated with treatment responsiveness and outcome. A different group recently analyzed the role of ADAMTS13 autoAbs in the caplacizumab era. The authors first identified a delay in the normalization of ADAMTS13 activity (>30%) in a subgroup of caplacizumab-treated patients, which was not evident in the pre-caplacizumab era [54]. The authors then evaluated the potential role of ADAMTS13 autoAbs levels and ADAMTS13 antigen in predicting the delayed normalization of ADAMTS13 activity in patients with an ADAMTS13 activity < 10% at the time of stopping caplacizumab. Presenting anti-ADAMTS13 IgG levels were not predictive of ADAMTS13 activity delayed normalization, yet a rise in autoAbs levels from diagnosis to the time of stopping caplacizumab appeared relevant. Furthermore, concurrent ADAMTS13 antigen levels < 30% at the time of caplacizumab discontinuation were associated with a greater risk of recurrence (defined in this study as any exacerbation or relapse). However, raised anti-ADAMTS13 IgG levels were not predictive of TTP recurrence. Our group, on a retrospective analysis of 42 first iTTP episodes, identified ADAMTS13 autoAbs titer at diagnosis as a marker of iTTP burden of care, associated with higher total number of PEX sessions, PEXs needed to achieve clinical response, days of hospitalization and a higher probability of needing RTX rescue to achieve clinical response [55]. In other words, ADAMTS13 autoAbs titer could identify those iTTP cases in which caplacizumab, currently the standard of care, can bring the greatest benefits compared with standard of care in terms of cost-effective analysis, and the cases in which early intensification of immunosuppressive with RTX is indicated.

While the significance of reduced ADAMTS13 activity during remission is more consolidated, the predictive value of disease recurrence determined by the presence of ADAMTS13 autoAbs, their inhibitory activity and Ig classes and subclasses is still controversial. In different studies, ADAMTS13 autoAbs during remission emerged as one of the possible risk factors associated with an increased risk of iTTP relapse, together with young age, race, a previous relapse of iTTP and severely deficient ADAMTS13 activity in remission [56,57,58]. Peyvandi and colleagues reported that the prevalence of any ADAMTS13 autoAbs (whether or not they inhibited protease activity) was significantly different in patients with or without recurrence. In their study, 64% of patients with recurrent iTTP had ADAMTS13 autoAbs during remission, whereas only 36% of those without recurrence had ADAMTS13 autoAbs. The unadjusted odds ratios for recurrence indicated that the presence of ADAMTS13 autoAbs, regardless of neutralizing activity, increased the likelihood of TTP recurrence by approximately three-fold [59]. These data were also supported by another study in which the presence of ADAMTS13 autoAbs during remission appeared to predict the risk of recurrence [47]. The cited study added that anti-ADAMTS13 IgG had a strong predictive value for recurrence during both acute and remission phases; no association between IgG subclasses and recurrence risk or association between ADAMTS13 autoAbs levels and acute episode severity was detected, in disagreement with previous reports [53]. An Italian group proposed with their analysis that ADAMTS13 activity ≤ 20%, ADAMTS13 autoAbs titer ≥ 15 U/mL and inhibitory activity > 50% at the time of remission correlated with disease relapse. The multivariate analysis showed that in the group of patients achieving a complete remission with PEX and steroids the combination of ADAMTS13 activity ≤ 20% with ADAMTS13 autoAbs ≥ 15 U/mL at remission and a time to response to first-line treatment ≥ 13 days, were independent predictive factors of relapse. During the follow-up, ADAMTS13 activity ≤ 20% and autoAbs titer ≥ 15 U/mL at 3 and 6 months were also associated with disease relapse; the combination of ADAMTS13 activity ≤ 20% with ADAMTS13 autoAbs ≥ 15 U/mL measured at 3 and 6 months was again identified as an independent predictor of disease relapse. Cox regression analysis indicated that patients with ADAMTS13 activity ≤ 20% plus ADAMTS13 autoAbs ≥ 15 U/mL at remission have an increased risk of relapse compared to patients with ADAMTS13 activity > 20% plus ADAMTS13 autoAbs < 15 U/mL [60]. However, not all authors agree with the prognostic role of ADAMTS13 inhibitors. For example, Jin and colleagues did not find any predictive value for IgG anti-ADAMTS13 levels measured during remission and the risk of recurrence [61]. Mancini et al. focused on a different marker, namely ADAMTS13-specific circulating immune complexes (CICs) [62]. The presence of circulating ADAMTS13s specific CICs in patients with iTTP has been reported both in acute and remission phases [27,28]. In their analysis, ADAMTS13-specific CICs of IgG isotype were not found to be markers of disease severity, but they were found to have a possible prognostic role as predictors of recurrence. This was especially true in the first two years after iTTP onset, with a 4.2-fold increased risk. A prospective study and a case series [63,64] report on pre-emptive treatment with RTX in patients with iTTP and persistent ADAMTS13 autoAbs, confirming the efficacy of such therapy, but currently there are no published studies or clinical trials registered aiming to establish antibody titer or a precise timepoint at which to consider pre-emptive therapy.

Summing up, together with ADAMTS13 activity and other clinical and laboratory factors, the role of ADAMTS13 autoAbs has been and is currently an interesting object of research. Even if global agreement between authors has not been reached, ADAMTS13 autoAbs are emerging as possible markers of a more severe acute form of iTTP, associated with a longer time to platelet count recovery, a higher number of PEX sessions, mortality and burden of care. Such conclusions could also be used to optimize the selection of those patients needing early immunosuppressive treatment intensification with RTX or, on the other hand, those with good chance to achieve clinical response with only steroid therapy. Furthermore, ADAMTS13 autoAbs together with ADAMTS13 activity may also represent a useful tool in the remission phase, able to identify patients with higher recurrence risk and with possible indication for pre-emptive treatment.

## 5. Conclusions

ADAMTS13 autoAbs represent only one of the interesting and not fully understood aspects of iTTP. Much progress in understanding the pathophysiological mechanisms has been made. Interest in such mechanisms is growing given the important clinical implications they may have with the advent of new therapeutic molecules. Their prognostic value is another important aspect. Although a unanimous consensus cannot be drawn by published studies, ADAMTS13 autoAbs appear to play a role both in the identification of more severe forms of acute iTTP and as a marker of recurrence risk during the remission phase. Thus, the refinement of laboratory methods aimed at their detection and characterization as well as large data collections will represent a cornerstone in the world of iTTP for the coming years.

## Data Availability

No new data were created or analyzed in this study. Data sharing is not applicable to this article.

## References

[1] Zheng X.L. (2015). ADAMTS13 and von Willebrand factor in thrombotic thrombocytopenic purpura. Annu. Rev. Med..

[2] Shin J.S., Subhan M.O., Cambridge G., Guo Y. (2022). Alterations in B- and circulating T-follicular helper cell subsets in immune thrombotic thrombocytopenic purpura. Blood Adv..

[3] Sorvillo N., van Haren S.D., Kaijen P.H., ten Brinke A. (2013). Preferential HLA-DRB1*11-dependent presentation of CUB2-derived peptides by ADAMTS13-pulsed dendritic cells. Blood.

[4] Verbij F.C., Turksma A.W., de Heij F., Kaijen P. (2016). CD4+ T cells from patients with acquired thrombotic thrombocytopenic purpura recognize CUB2 domain-derived peptides. Blood.

[5] Carsetti R., Rosado M.M., Wardmann H. (2004). Peripheral development of B cells in mouse and man. Immunol. Rev..

[6] Sitaru C., Mihai S., Zillikens D. (2007). The relevance of the IgG subclass of autoantibodies for blister induction in autoimmune bullous skin diseases. Arch. Dermatol. Res..

[7] Maran R., Dueymes M., Le Corre R., Renaudineau Y., Shoenfeld Y. (1997). IgG subclasses of human autoantibodies. Ann. Med. Interne.

[8] Ferrari S., Mudde G.C., Rieger M., Veyradier A. (2009). IgG subclass distribution of anti-ADAMTS13 antibodies in patients with acquired thrombotic thrombocytopenic purpura. J. Thromb. Haemost..

[9] Zheng X., Chung D., Takayama T.K., Majerus E.M. (2001). Structure of von Willebrand factor-cleaving protease (ADAMTS13), a metalloprotease involved in thrombotic thrombocytopenic purpura. J. Biol. Chem..

[10] South K., Luken B.M., Crawley J.T., Phillips R. (2014). Conformational activation of ADAMTS13. Proc. Natl. Acad. Sci. USA.

[11] Roose E., Schelpe A.S., Tellier E., Sinkovits G. (2020). Open ADAMTS13, induced by antibodies, is a biomarker for subclinical immune-mediated thrombotic thrombocytopenic purpura. Blood.

[12] Roose E., Vidarsson G., Kangro K., Verhagen O.J.H.M. (2018). Anti-ADAMTS13 Autoantibodies against Cryptic Epitopes in Immune-Mediated Thrombotic Thrombocytopenic Purpura. Thromb. Haemost..

[13] De Waele L., Curie A., Kangro K., Tellier E. (2021). Anti-cysteine/spacer antibodies that open ADAMTS13 are a common feature in iTTP. Blood Adv..

[14] Luken B.M., Turenhout E.A., Hulstein J.J., Van Mourik J.A. (2005). The spacer domain of ADAMTS13 contains a major binding site for antibodies in patients with thrombotic thrombocytopenic purpura. Thromb. Haemost..

[15] Luken B.M., Kaijen P.H., Turenhout E.A., Kremer Hovinga J.A. (2006). Multiple B-cell clones producing antibodies directed to the spacer and disintegrin/thrombospondin type-1 repeat 1 (TSP1) of ADAMTS13 in a patient with acquired thrombotic thrombocytopenic purpura. J. Thromb. Haemost..

[16] Yamaguchi Y., Moriki T., Igari A., Nakagawa T. (2011). Epitope analysis of autoantibodies to ADAMTS13 in patients with acquired thrombotic thrombocytopenic purpura. Thromb. Res..

[17] Velásquez Pereira L.C., Roose E., Graça N.A.G., Sinkovits G. (2021). Immunogenic hotspots in the spacer domain of ADAMTS13 in immune-mediated thrombotic thrombocytopenic purpura. J. Thromb. Haemost..

[18] Klaus C., Plaimauer B., Studt J.D., Dorner F. (2004). Epitope mapping of ADAMTS13 autoantibodies in acquired thrombotic thrombocytopenic purpura. Blood.

[19] Ostertag E.M., Kacir S., Thiboutot M., Gulendran G. (2016). ADAMTS13 autoantibodies cloned from patients with acquired thrombotic thrombocytopenic purpura: 1. Structural and functional characterization in vitro. Transfusion.

[20] Thomas M.R., de Groot R., Scully M.A., Crawley J.T. (2015). Pathogenicity of Anti-ADAMTS13 Autoantibodies in Acquired Thrombotic Thrombocytopenic Purpura. EBioMedicine.

[21] Kangro K., Roose E., Joly B.S., Sinkovits G. (2021). Anti-ADAMTS13 autoantibody profiling in patients with immune-mediated thrombotic thrombocytopenic purpura. Blood Adv..

[22] Sakai K., Matsumoto M., De Waele L., Dekimpe C. (2023). ADAMTS13 conformation and immunoprofiles in Japanese patients with immune-mediated thrombotic thrombocytopenic purpura. Blood Adv..

[23] Graça N.A.G., Ercig B., Carolina Velasquez Pereira L., Kangro K. (2020). Modifying ADAMTS13 to modulate binding of pathogenic autoantibodies of patients with acquired thrombotic thrombocytopenic purpura. Haematologica.

[24] Jian C., Xiao J., Gong L., Skipwith C.G., Jin S.Y. (2012). Gain-of-function ADAMTS13 variants that are resistant to autoantibodies against ADAMTS13 in patients with acquired thrombotic thrombocytopenic purpura. Blood.

[25] Soejima K., Matsumoto M., Kokame K., Yagi H. (2003). ADAMTS-13 cysteine-rich/spacer domains are functionally essential for von Willebrand factor cleavage. Blood.

[26] Ferrari S., Knöbl P., Kolovratova V., Plaimauer B. (2012). Inverse correlation of free and immune complex-sequestered anti-ADAMTS13 antibodies in a patient with acquired thrombotic thrombocytopenic purpura. J. Thromb. Haemost..

[27] Ferrari S., Palavra K., Gruber B., Kremer Hovinga J.A., Knöbl P., Caron C., Cromwell C., Aledort L., Plaimauer B., Turecek P.L. (2014). Persistence of circulating ADAMTS13-specific immune complexes in patients with acquired thrombotic thrombocytopenic purpura. Haematologica.

[28] Lotta L.A., Valsecchi C., Pontiggia S., Mancini I. (2014). Measurement and prevalence of circulating ADAMTS13-specific immune complexes in autoimmune thrombotic thrombocytopenic purpura. J. Thromb. Haemost..

[29] Vugmeyster Y., Xu X., Theil F.P., Khawli L.A. (2012). Pharmacokinetics and toxicology of therapeutic proteins: Advances and challenges. World J. Biol. Chem..

[30] Schifferli J.A., Taylor R.P. (1989). Physiological and pathological aspects of circulating immune complexes. Kidney Int..

[31] Emlen W., Carl V., Burdick G. (1992). Mechanism of transfer of immune complexes from red blood cell CR1 to monocytes. Clin. Exp. Immunol..

[32] Johansson A., Erlandsson A., Eriksson D., Ullén A. (2002). Idiotypic-anti-idiotypic complexes and their in vivo metabolism. Cancer.

[33] Kosugi I., Muro H., Shirasawa H., Ito I. (1992). Endocytosis of soluble IgG immune complex and its transport to lysosomes in hepatic sinusoidal endothelial cells. J. Hepatol..

[34] Underwood M.I., Alwan F., Thomas M.R., Scully M.A. (2023). Autoantibodies enhance ADAMTS-13 clearance in patients with immune thrombotic thrombocytopenic purpura. J. Thromb. Haemost..

[35] Smock K.J. (2021). ADAMTS13 testing update: Focus on laboratory aspects of difficult thrombotic thrombocytopenic purpura diagnoses and effects of new therapies. Int. J. Lab. Hematol..

[36] Page E.E., Kremer Hovinga J.A., Terrell D.R., Vesely S.K. (2017). Thrombotic thrombocytopenic purpura: Diagnostic criteria, clinical features, and long-term outcomes from 1995 through 2015. Blood Adv..

[37] Hubbard A.R., Heath A.B., Kremer Hovinga J.A. (2015). Subcommittee on von Willebrand Factor. Establishment of the WHO 1st International Standard ADAMTS13, plasma (12/252): Communication from the SSC of the ISTH. J. Thromb. Haemost..

[38] George J.N. (2018). The remarkable diversity of thrombotic thrombocytopenic purpura: A perspective. Blood Adv..

[39] Peyvandi F., Palla R., Lotta L.A., Mackie I. (2010). ADAMTS-13 assays in thrombotic thrombocytopenic purpura. J. Thromb. Haemost..

[40] Moore G.W., Vetr H., Binder N.B. (2023). ADAMTS13 Antibody and Inhibitor Assays. Methods Mol. Biol..

[41] Vendramin C., Thomas M., Westwood J.P., Scully M. (2018). Bethesda Assay for Detecting Inhibitory Anti-ADAMTS13 Antibodies in Immune-Mediated Thrombotic Thrombocytopenic Purpura. TH Open.

[42] Kremer Hovinga J.A., Heeb S.R., Skowronska M., Schaller M. (2018). Pathophysiology of thrombotic thrombocytopenic purpura and hemolytic uremic syndrome. J. Thromb. Haemost..

[43] Rieger M., Mannucci P.M., Kremer Hovinga J.A., Herzog A. (2005). ADAMTS13 autoantibodies in patients with thrombotic microangiopathies and other immunomediated diseases. Blood.

[44] Shelat S.G., Ai J., Zheng X.L. (2005). Molecular biology of ADAMTS13 and diagnostic utility of ADAMTS13 proteolytic activity and inhibitor assays. Semin. Thromb. Hemost..

[45] Dekimpe C., Roose E., Kangro K., Bonnez Q. (2021). Determination of anti-ADAMTS-13 autoantibody titers in ELISA: Influence of ADAMTS-13 presentation and autoantibody detection. J. Thromb. Haemost..

[46] Masias C., Cataland S.R. (2018). The role of ADAMTS13 testing in the diagnosis and management of thrombotic microangiopathies and thrombosis. Blood.

[47] Bettoni G., Palla R., Valsecchi C., Consonni D. (2012). ADAMTS-13 activity and autoantibodies classes and subclasses as prognostic predictors in acquired thrombotic thrombocytopenic purpura. J. Thromb. Haemost..

[48] Roos A., Bouwman L.H., van Gijlswijk-Janssen D.J., Faber-Krol M.C. (2001). Human IgA activates the complement system via the mannan-binding lectin pathway. J. Immunol..

[49] Zheng X.L., Kaufman R.M., Goodnough L.T., Sadler J.E. (2004). Effect of plasma exchange on plasma ADAMTS13 metalloprotease activity, inhibitor level, and clinical outcome in patients with idiopathic and nonidiopathic thrombotic thrombocytopenic purpura. Blood.

[50] Vesely S.K., George J.N., Lämmle B., Studt J.D. (2003). ADAMTS13 activity in thrombotic thrombocytopenic purpura-hemolytic uremic syndrome: Relation to presenting features and clinical outcomes in a prospective cohort of 142 patients. Blood.

[51] Veyradier A., Obert B., Houllier A., Meyer D. (2001). Specific von Willebrand factor-cleaving protease in thrombotic microangiopathies: A study of 111 cases. Blood.

[52] Alwan F., Vendramin C., Vanhoorelbeke K., Langley K. (2017). Presenting ADAMTS13 antibody and antigen levels predict prognosis in immune-mediated thrombotic thrombocytopenic purpura. Blood.

[53] Coppo P., Wolf M., Veyradier A., Bussel A. (2006). Réseau d’Etude des Microangiopathies Thrombotiques de l’Adulte. Prognostic value of inhibitory anti-ADAMTS13 antibodies in adult-acquired thrombotic thrombocytopenic purpura. Br. J. Haematol..

[54] Prasannan N., Thomas M., Stubbs M., Westwood J.P. (2023). Delayed normalization of ADAMTS13 activity in acute thrombotic thrombocytopenic purpura in the caplacizumab era. Blood.

[55] Dainese C., Valeri F., Pizzo E., Valpreda A. (2022). ADAMTS13 Autoantibodies and Burden of Care in Immune Thrombotic Thrombocytopenic purpura: New Evidence and Future Implications. Clin. Appl. Thromb. Hemost..

[56] Jestin M., Benhamou Y., Schelpe A.S., Roose E. (2018). French Thrombotic Microangiopathies Reference Center. Preemptive rituximab prevents long-term relapses in immune-mediated thrombotic thrombocytopenic purpura. Blood.

[57] Mai Falk J., Scharrer I. (2016). Idiopathic thrombotic thrombocytopenic purpura: Strongest risk factor for relapse from remission is having had a relapse. Transfusion.

[58] Liu A., Mazepa M., Davis E., Johnson A., Antun A.G., Farland A.M., Woods R.R., Metjian A., Bagby K., Park Y. (2019). African American race is associated with decreased relapse-free survival in immune thrombotic thrombocytopenic purpura. Blood.

[59] Peyvandi F., Lavoretano S., Palla R., Feys H.B. (2008). ADAMTS13 and anti-ADAMTS13 antibodies as markers for recurrence of acquired thrombotic thrombocytopenic purpura during remission. Haematologica.

[60] Schieppati F., Russo L., Marchetti M., Barcella L. (2020). Low levels of ADAMTS-13 with high anti-ADAMTS-13 antibodies during remission of immune-mediated thrombotic thrombocytopenic purpura highly predict for disease relapse: A multi-institutional study. Am. J. Hematol..

[61] Jin M., Casper T.C., Cataland S.R., Kennedy M.S. (2008). Relationship between ADAMTS13 activity in clinical remission and the risk of TTP relapse. Br. J. Haematol..

[62] Mancini I., Ferrari B., Valsecchi C., Pontiggia S. (2017). Italian Group of TTP Investigators. ADAMTS13-specific circulating immune complexes as potential predictors of relapse in patients with acquired thrombotic thrombocytopenic purpura. Eur. J. Intern. Med..

[63] Fakhouri F., Vernant J.P., Veyradier A., Wolf M. (2005). Efficiency of curative and prophylactic treatment with rituximab in ADAMTS13-deficient thrombotic thrombocytopenic purpura: A study of 11 cases. Blood.

[64] Bresin E., Gastoldi S., Daina E., Belotti D. (2009). Rituximab as pre-emptive treatment in patients with thrombotic thrombocytopenic purpura and evidence of anti-ADAMTS13 autoantibodies. Thromb. Haemost..

